# Special Issue: Iconic Rice Varieties

**DOI:** 10.1186/s12284-018-0214-5

**Published:** 2018-04-09

**Authors:** David J. Mackill

**Affiliations:** Mars, Inc., Department of Plant Sciences, University of California, Davis, CA 95618 USA

The 50th anniversary of the November 1966 release of the rice variety IR8 as the “world’s first modern rice variety” was recently celebrated at IRRI headquarters (http://irri.org/ir8). The variety was based on a high-yielding, semidwarf plant ideotype (Jennings 1964). IR8 provided a huge yield advantage when combined with improved agronomic management practices, but it had significant defects, such as poor cooking quality, long-duration, and susceptibility to diseases and insects. Nevertheless, paying tribute to such impactful rice varieties should be more widely practiced.

Through the established methods of plant breeding, numerous varieties with improved yield, adaptation, resistance to diseases and insects, and grain quality have been developed and disseminated to farmers, especially in Asia. Many of these varieties became great successes and were grown on larger areas over appreciable time periods, providing huge benefits to farmers and consumers. In addition, they have become the varieties of reference for scientific studies ranging from genomics and plant biology to improved cultivation methods for rice. However, the story of their development is not well documented in the literature.

This special issue of *Rice* reviews the development of five of these iconic varieties. The varieties described are very different. The Hom Mali variety of Thailand (Vanavichit et al. 2018) and Koshihikari of Japan (Kobayashi et al. 2018) are from the pre-IR8 era and do not share its semidwarf plant type. Both of these varieties are renowned for their premium quality and are sold internationally. Pusa Basmati 1121 is also widely celebrated for grain quality, especially its extraordinary grain elongation upon cooking, but it is more clearly a modern variety with semidwarf plant type (Singh et al. 2018). IR64 has been described as the world’s most popular variety; it is a high-yielding indica rice and also has good cooking quality (Mackill and Khush 2018). Finally, Shanyou 63 is historically the most widely-grown hybrid rice variety and has contributed greatly to food production and the further development of hybrid rice in China (Xie and Zhang 2018).

Rice breeders normally carry a large number of fixed lines up to an advanced stage of testing and hope to release the best of them as new varieties. They usually cannot predict the success of any variety until it reaches the farmers, who try them out and decide which ones to keep growing in subsequent seasons. The grains of the new variety must also be acceptable to the rice buyers, millers and consumers. Among the many varieties released, a few have much greater success, and the area of their cultivation increases. It is difficult for the seed system to maintain a larger number of varieties than to maintain one or two varieties, so once a mega variety becomes dominant there is a decline in the number of varieties grown. This is one reason there is inertia to adopt new varieties, unless there will be a strong demand from the producers and the industry and consumers. Also, while farmers in developed countries change varieties frequently, farmers in many rice-growing countries grow older varieties, often released over 15 years ago (Witcombe et al. 2017).

Rice breeders must build on these varieties and others for further improvements in important traits, especially grain yield. Trait introgression through marker assisted backcrossing is a common approach for adding value to these varieties. While it has limitations as far as how much improvement can be made, it remains a viable strategy of rapid gene delivery for rice as it does for other important crops (Cameron et al. 2017). Modern breeding approaches such as genetic engineering and gene editing can be used to modify these varieties for specific traits.

Breeders must continue to develop novel, superior varieties that can replace these varieties and provide more benefits to the farmers and rice consumers. Although this is a challenging objective, it is encouraging that some of the varieties covered in this issue have been or are in the process of being replaced with new varieties. Genomic selection may have a role to play in assembling a larger number of favorable alleles into a new variety (Hickey et al. 2017). As more information on the genetic control of traits becomes available, breeders will be able to select for the best alleles combining multiple traits in new varieties. A recent example is the use of markers for multiple genes controlling yield and quality in a complex gene pyramiding or “rational design” approach (Zeng et al. 2017).

These important rice varieties are the foundation for the current generation of rice breeders to develop new varieties that exceed them in performance. They have made major contributions to the economies of rice-growing countries. It is necessary to document these successes. Many other important varieties could have been covered in this special issue. It is hoped that their stories will be written by other authors.
